# TCR-H: explainable machine learning prediction of T-cell receptor epitope binding on unseen datasets

**DOI:** 10.3389/fimmu.2024.1426173

**Published:** 2024-08-16

**Authors:** Rajitha Rajeshwar T., Omar N. A. Demerdash, Jeremy C. Smith

**Affiliations:** ^1^ UT/ORNL Center for Molecular Biophysics, Oak Ridge National Laboratory, Oak Ridge, TN, United States; ^2^ Department of Biochemistry and Cellular and Molecular Biology, University of Tennessee, Knoxville, TN, United States; ^3^ Biosciences Division, Oak Ridge National Laboratory, Oak Ridge, TN, United States

**Keywords:** T-cell receptor, epitope, antigen, explainable machine learning, physicochemical model, adaptive immunity T-cell receptor, machine learning, physicochemical features

## Abstract

Artificial-intelligence and machine-learning (AI/ML) approaches to predicting T-cell receptor (TCR)-epitope specificity achieve high performance metrics on test datasets which include sequences that are also part of the training set but fail to generalize to test sets consisting of epitopes and TCRs that are absent from the training set, i.e., are ‘unseen’ during training of the ML model. We present TCR-H, a supervised classification Support Vector Machines model using physicochemical features trained on the largest dataset available to date using only experimentally validated non-binders as negative datapoints. TCR-H exhibits an area under the curve of the receiver-operator characteristic (AUC of ROC) of 0.87 for epitope ‘hard splitting’ (i.e., on test sets with all epitopes unseen during ML training), 0.92 for TCR hard splitting and 0.89 for ‘strict splitting’ in which neither the epitopes nor the TCRs in the test set are seen in the training data. Furthermore, we employ the SHAP (Shapley additive explanations) eXplainable AI (XAI) method for *post hoc* interrogation to interpret the models trained with different hard splits, shedding light on the key physiochemical features driving model predictions. TCR-H thus represents a significant step towards general applicability and explainability of epitope:TCR specificity prediction.

## Introduction

Cytotoxic T-cells are central to the adaptive immune response. Critical to adaptive immune system activation is the specific binding of T-cell receptors (TCR) to peptide epitopes presented by the Major Histocompatibility Complex (MHC) on the surface of antigen-presenting cells. The remarkable diversity of TCRs, estimated at ~10^15^-10^61^ (1), results from a combinatorial explosion of genetic recombination possibilities of somatic-cell DNA encoding V (variable), D (diversity), and J (joining) segments (2, 3). T-cells are thus capable of recognizing a large variety of epitopes, that can be exogenous, such as from a pathogen, or derived from endogenous mutated proteins (4).

A significant goal in immunology is the reliable prediction of which epitopes bind to which T-cell receptors. Achieving this will greatly aid in the design and development of vaccines and immunotherapies and will help us understand how the immune system distinguishes between self and non-self-antigens. Furthermore, knowledge of TCR:epitope specificity can be used in disease diagnosis, prognosis, and monitoring disease progression, for example in the context of infectious diseases, where tracking T-cell responses can provide insights into the immune system’s response to pathogens.

The vast diversity in TCR sequences and potential epitopes makes it challenging to develop a generalized and accurate computational model for TCR:epitope binding prediction. Moreover, prediction is further complicated by the fact that TCRs can exhibit cross-reactivity, recognizing multiple epitopes. One approach, in principle, to solving the TCR:epitope specificity problem would be to develop accurate methods for predicting the 3D structures of TCR:epitope:MHC ternary complexes and then to use the results to predict binding strength based on the physics involved. However, predicting these 3D structures and interactions accurately is challenging (5), as the 3D interactions of TCRs with epitopes bound to MHC are highly variable and can be significantly impacted by small changes in epitope sequence. Furthermore, the binding of the ternary complex is very weak, in the micromolar range, limiting both experimental and first-principles-physics-based models (6, 7). Conformational flexibility of TCRs further complicates this approach. Moreover, aside from the interactions within the ternary structure in isolation, the binding in the ternary structure is mechanosensitive, whereby the affinity is further modulated by forces exerted on T-cells during motility processes (8).

In recent work we demonstrated how 3D structure can be incorporated into computational methods to improve prediction of peptide binding to MHC (9), and how 3D MHC pocket structural similarity correlates with the ability to bind to a given peptide with similar binding affinity (10). Furthermore, we and others have shown that certain 3D interactions are commonly found in epitope binding to TCRs (3, 11–14). We therefore postulated that TCR:epitope recognition algorithms would benefit from consideration of physicochemical properties determining recognition.

TCRs are heterodimers consisting of two chains: α and β. Binding primarily occurs through three complementarity-determining regions (CDRs) found on each of these chains. Although in 3D structures of TCR:epitope:MHC ternary complexes significant contacts are made outside the CDR3 region, this region most directly interacts with the peptide epitope, with CDR3β, in particular, forming numerous direct contacts with the epitopes and MHC while also displaying particularly high sequence diversity (11, 12, 15–17). Hence, in computational approaches CDR3β has commonly been taken as an approximation to the specificity-determining TCR sequence.

With the increasing availability in publicly available data resources of CDR3β and epitope sequencing data from high-throughput techniques (18), AI and ML approaches have been able to be used to predict TCR CDR3β binding to epitopes presented by MHC class 1 (MHC-I) (19). These tools apply methods ranging from relatively simple ML algorithms such as Random Forest and clustering (1, 18, 20, 21) to various forms of deep learning-based AI techniques, including convolutional and recurrent neural networks (18, 22–30).

Many existing AI/ML methods achieve impressive results on “random split” test sets that include TCR and epitope sequences that are also included in the training set. However, for general applicability a model must be able to predict binding for a “strict split” with unseen TCRs and epitopes, i.e., in which neither the TCRs nor the epitopes tested are in the training set. Success in this endeavor would perhaps indicate an algorithm has been discovered that has started to learn the general principles of TCR:epitope recognition and would be a major step towards a “holy grail” of immunology - the accurate computational mapping of all TCRs to their cognate epitopes.

The strict split goal can be approached stepwise. In a first step, datasets can be derived with an epitope “hard split”, i.e., in which the test set contains only unseen epitopes, or a TCR hard split in which the test set contains only unseen CDR3βs. When using an epitope hard split, current methods mostly fail, with scores falling to almost random i.e., with the area under the curve of the receiver-operator characteristic (AUC of ROC) ~0.5 (31, 32).. TCR hard splitting has been performed in (23) with some success, with AUC values of 0.77 or below. Strict splitting attempts have also been published with AUC of ROC values ranging between 0.5 and 0.7 (30, 33).

Given the above considerations, we attempted to address the problem of unseen epitopes and TCRs by developing a supervised binary classification ML model incorporating sequence-based physicochemical descriptors of CDR3β and epitopes and using the largest dataset of binding and non-binding data available to date. Our approach possesses two particularly noteworthy characteristics: the use of only experimentally validated non-binders as negative datapoints in the training and testing sets and the use of a highly diverse set of physicochemical features calculated over entire peptide sequences.

Existing machine and deep learning methods vary in terms of the datasets used, mostly relying on randomly generated data as the negative/non-binding dataset. However, any given randomly generated TCR: CDR3β sequence pair assumed to not bind might actually bind. Furthermore, it has been shown that the use of randomly generated sequences for negative binders leads to overestimation of model accuracy (31, 32). Therefore, we include in our dataset only negatives that have been experimentally validated as non-binders.

The choice of supervised ML over deep learning was motivated in part by the ability of the model to be based on explicit features posited to be important for binding, which in turn may aid model explainability and rationality. While understanding ML model decisions is not always straightforward, advancements in eXplainable AI (XAI) methods enable us to pinpoint the contributions of input features and identify those that influence model predictions. Moreover, XAI offers insights into how the model can be enhanced. Additionally, our preference for ML was informed by previous observations suggesting that ML methods outperform deep learning methods in molecular property predictions when well-defined features are employed (34), and it has been shown that the use of features that lend themselves to ML models leads to better performance in TCR specificity prediction (21).

Most ML models have hitherto been sequence-based, encoding sequences using either the BLOSUM substitution matrix (21, 23–25) and/or Atchley factors (35). The BLOSUM matrix applies a score for amino-acid substitutions while Atchley factors are multidimensional, composite features for each single amino acid independently, derived using unsupervised ML on primarily physicochemical features (26, 36, 37). Here, in contrast we use physicochemical features calculated over entire epitope and CDR3β sequences i.e., not just individual amino-acid residues.

Various ML methods are tested: Random Forest (RF), Gradient Boosting trees (GBT), eXtreme Gradient Boosting (xGBT) and Support Vector Machines (SVM). The best performing, an SVM model, which we name TCR-H, exhibited area under the receiver-operator characteristic curve (AUC of ROC) metrics of 0.87, 0.92 and 0.89 for the epitope hard split, TCR hard split and the epitope/TCR strict split, respectively. These are significant improvements on previously reported values, and TCR-H thus represents a noteworthy step towards general applicability of computational prediction of epitope:TCR specificity in biology and medicine.

## Materials and methods

### Dataset

The dataset used comprises positive binding data curated for human MHC class 1 from the IEDB, VDJdb and McPAS-TCR databases and negative binding data from IEDB and 10X Genomics assays, sourced from TChard (32). The dataset includes CDR3β sequence lengths ranging from 9 to 23 residues, while the epitope sequences have lengths shorter than 16. The total dataset consists of 147,069 negative and 107,376 positive datapoints and was divided into various training and test data sets of 80 and 20 percent, respectively.

Two different types of data split were considered: 1) Hard Split 2) Random Split. In the epitope hard split the test set consisted of only unseen epitopes. The epitope hard split training dataset (total: 200,011) consisted of 124,598 negative data and 75,413 positive datapoints, while the test dataset (total: 54,434) consisted of 22,471 negative data and 31,963 positive data points, with 65 unseen epitopes. For the TCR hard split, again the training and test data were split approximately in the ratio of 80:20 with the training set consisting of 117,523 negative and 85,621 positive data points, while in the test dataset there were 21,754 positive and 29,546 negative data. In the strict split, the training data consisted of 65826 positive data and 83697 negative data points whereas test dataset consisted of 22,471 negative data and 31,963 positive data. The training and test data sets are detailed in the supporting information (**Supplementary Table 1**).

### Feature set

All sequence-based properties of the CDR3β loops and the epitopes were calculated using the Peptides Python package [https://github.com/althonos/peptides.py] (38), resulting in 96 features each for the CDR3β and epitope sequences. A complete list of the features used is given in **Table 1**.

**Table 1:** Sequence-based properties used as features (96 each for epitope and CDR3β) in the present study are tabulated.

Sequence-based property	Number of features
BLOSUM indices	10
Cruciani properties	8
FASGAI vectors	6
Kidera factors	10
MS-WHIM scores	3
PCP descriptors	5
Physical_descriptors	2
ProtFP descriptors	8
Sneath vectors	4
SVGER_descriptors	10
ST-scales	8
T-scales	5
VHSE-scales	8
Z-scales	5
Boman	1
Charge	1
Hydrophobic Moment	1
Hydrophobicity	1
Isoelectric Point	1
Molecular Weight	1
m/z(mass/charge)	1

The features used are now very briefly outlined below.

*BLOSUM Indices (BLOSUM 1-10):* These are derived by using the AAindex database and decomposing the BLOSUM62 substitution matrix into scales satisfying the VARIMAX criterion (39).

*Cruciani properties*: These properties are derived from scaled principal component scores, which encapsulate various descriptors reflecting the interaction of each amino-acid residue with different chemical groups. The average of the Cruciani properties (PP1, PP2, PP3) is calculated for all residues within the peptide sequence (40).

*FASGAI vectors:* FASGAI (Factor Analysis Scales of Generalized Amino Acid Information) vectors are six numerical amino acid descriptors that reflect hydrophobicity (F1), alpha and turn propensities (F2), bulkiness (F3), compositional characteristics (F4), local flexibility (F5), and electronic properties (F6) of the sequence (41).

*Kidera factors:* These are derived by applying multivariate analysis to 188 physical properties of the 20 amino acids, utilizing principal component analysis and factor analysis to reduce the dimensionality of the features. The average of the ten Kidera factors for a given protein sequence is obtained where the first four represent purely physical properties. In contrast, the remaining six factors are combinations of multiple physical properties, labeled for convenience with the name of the most heavily weighted component: KF1: helix/bend preference, KF2: side-chain size, KF3: extended structure preference, KF4: hydrophobicity, KF5: double-bend preference, KF6: partial specific volume, KF7: flat extended preference, KF8: occurrence in alpha region, KF9: pK-C, KF10: surrounding hydrophobicity (42).

*MSWHIM scales*: These scales use three components from a PCA (principal component analysis) of 3D electrostatic properties to represent residues. Component 1 (MSWHIM1) separates positive/aromatic from negative/bulkier residues. Component 2 (MSWHIM2) differentiates Asp/Glu, and component 3 (MSWHIM3) distinguishes Arg/Lys (43).

*PCP descriptors:* These are constructed by performing multidimensional scaling of 237 physicochemical properties (44, 45).

*Physical Descriptors:* Physical descriptors were constructed by improving on existing PCA-derived descriptors (Z-scales, MS-WHIM and T-scales) after correcting for the hydrophilicity of methionine, asparagine and tryptophan. *PD1* is related to volume while *PD2* is related to hydrophilicity (46, 47).

*ProtFP descriptors:* This descriptor set was formulated using an extensive compilation of indices sourced from the AAindex database for all naturally occurring amino acids (48, 49).

*Sneath vectors:* These vectors were obtained by running PCA on the ϕ coefficient to explain the dissimilarity between the 20 natural amino acids based on binary state encoding of 134 physical and chemical properties (such as presence/absence of a —CH₃ group, step-wise optical rotation, etc) (50).

*SVGER descriptors:* These descriptors were constructed by principal component analysis of 74 geometrical descriptors (svger1 to svger6), 44 eigenvalue descriptors (svger7, svger8 and svger9), and 41 Randić descriptors (svger10 and svger11) (51, 52).

*ST-scales:* These scales are obtained by consideration of 827 properties that primarily include constitutional, topological, geometrical, hydrophobic, electronic, and steric properties of a total set of 167 amino acids (53).

*T-scales:* These rely on 67 shared topological descriptors derived from 135 amino acids. These descriptors stem solely from the connectivity table of amino acids (54).

*VHSE scales:* These were generated through principal component analysis (PCA) of 50 physicochemical variables representing the 20 amino acids. These variables encompassed 18 hydrophobic properties, 17 steric properties, and 15 electronic properties, each treated as independent families. Specifically, VHSE1 and VHSE2 pertain to hydrophobic properties, VHSE3 and VHSE4 to steric properties, and VHSE5 to VHSE8 to electronic properties (55).

*Z-scales:* These are based on physicochemical properties of the residues including NMR and thin-layer chromatography (TLC) data. Each Z scale represents specific properties. Z1: lipophilicity, Z2: steric bulk and polarizability, Z3: electronic properties (polarity/charge), Z4 and Z5 relate electronegativity, heat of formation, electrophilicity and hardness (56).

*Boman Index:* This index sums the solubility values for all residues in the sequence and provides an estimate of the peptide’s potential to bind to membranes or other proteins as receptors. To normalize the index, it is divided by the number of residues in the sequence (57).

*Hydrophobic moment:* This utilizes the standardized Eisenberg scale (58).

*Hydrophobicity:* The hydrophobicity of the entire sequence is obtained by summing the hydrophobicities of individual amino acids and dividing by the length of the sequence (38).

*Instability index:* This indicates the stability of a protein based on its amino acid composition (59)

*Molecular weight:* This is weighted with ExPASy’s “compute pI/mw” tool (60).

*Isoelectric point:* It is the pH of the sequence at which the net charge of the sequence is equal to zero (61).

*m/z:* The ratio of mass to charge.

Some features differ substantially in magnitude from one another. In training ML models whose optimization is gradient-based, the presence of features differing by an order of magnitude or more can pose a numerical challenge and yield erroneous results. To avoid this problem, features were normalized, or scaled, so that they were all within the same order of magnitude. To achieve this, the *preprocessing.scale* function in Scikit Learn was used, which centers features to the mean and normalizes them to unit variance.

### Machine learning methods

We tested four ML methods: Random Forest (RF), Gradient Boosting trees (GBT), eXtreme Gradient Boosting, and Support Vector Machines (SVM). All models were trained using the Scikit Learn application programming interface.

Random Forest (RF) is an ensemble learning method, *i.e.*, in which the final model is a composite of multiple individual models. RF operates by constructing multiple simple decision trees during training. Each tree in the forest is constructed using a random subset of the features. The final prediction is based on the aggregated votes of individual trees. Here, the RF model was built using default hyperparameters and scaled features. Gradient-Boosting Trees (GBT) is another ensemble method. In contrast to RF, whose trees are constructed independently of one another during model training, GBT successively augments the model in each iteration with a tree such that it minimizes a loss function representing the discrepancy between the training targets and the corresponding model predictions. eXtreme Gradient Boosting (XGB) is similar to GBT in that it is based on minimizing a loss function with each tree that is successively added to the ensemble of trees, but instead of optimizing using just the gradient it makes use of the Hessian as well, in effect optimizing using a Newton-Raphson type of approach.

In classification, SVM aims to find the hyperplane that best separates the data into different classes, maximizing the margin between the classes. SVM can be trained on the original set of features (corresponding to a linear SVM model) or a projection of the original set of features into a non-linear space using different kernel functions. We used the Radial Basis Function (RBF) kernel, also known as the Gaussian kernel, with the default hyperparameters.

All the prediction models were built with scaled features and default hyperparameters. The use of default parameters does not require hyperparameter optimization via cross-validation.

### Explainable machine learning: SHAP

Explainable machine learning is a powerful technique that aids in understanding the decision-making process of any given ML model, thereby interpreting its predictions. The interpretability and explainability of a model allow us to discern which features are important and to what extent each feature contributes to the model’s predictions. In this study, we utilized SHapley Additive exPlanations (SHAP), an approach to interrogate ML models *post hoc* that employs Shapley values and cooperative game theory (62). We opted for SHAP over other approaches, such as LIME (63) and permutation importance, because it is model-agnostic and offers both global and local interpretability. This means that SHAP can elucidate how features contribute to predictions across the entire dataset or for a single prediction, unlike LIME, which only provides information on a region of feature-ML target space, providing a linear model proxy in that region. The Python SHAP package was utilized for KernelSHAP calculations, with the n_samples parameter, controlling the number of Monte Carlo samples used for approximation, set to 100. A summary plot of SHAP values for all features across different subsets of the hard split and random split test datasets was generated to visualize the important features contributing to binding prediction.

## Results

**Figure 1:**
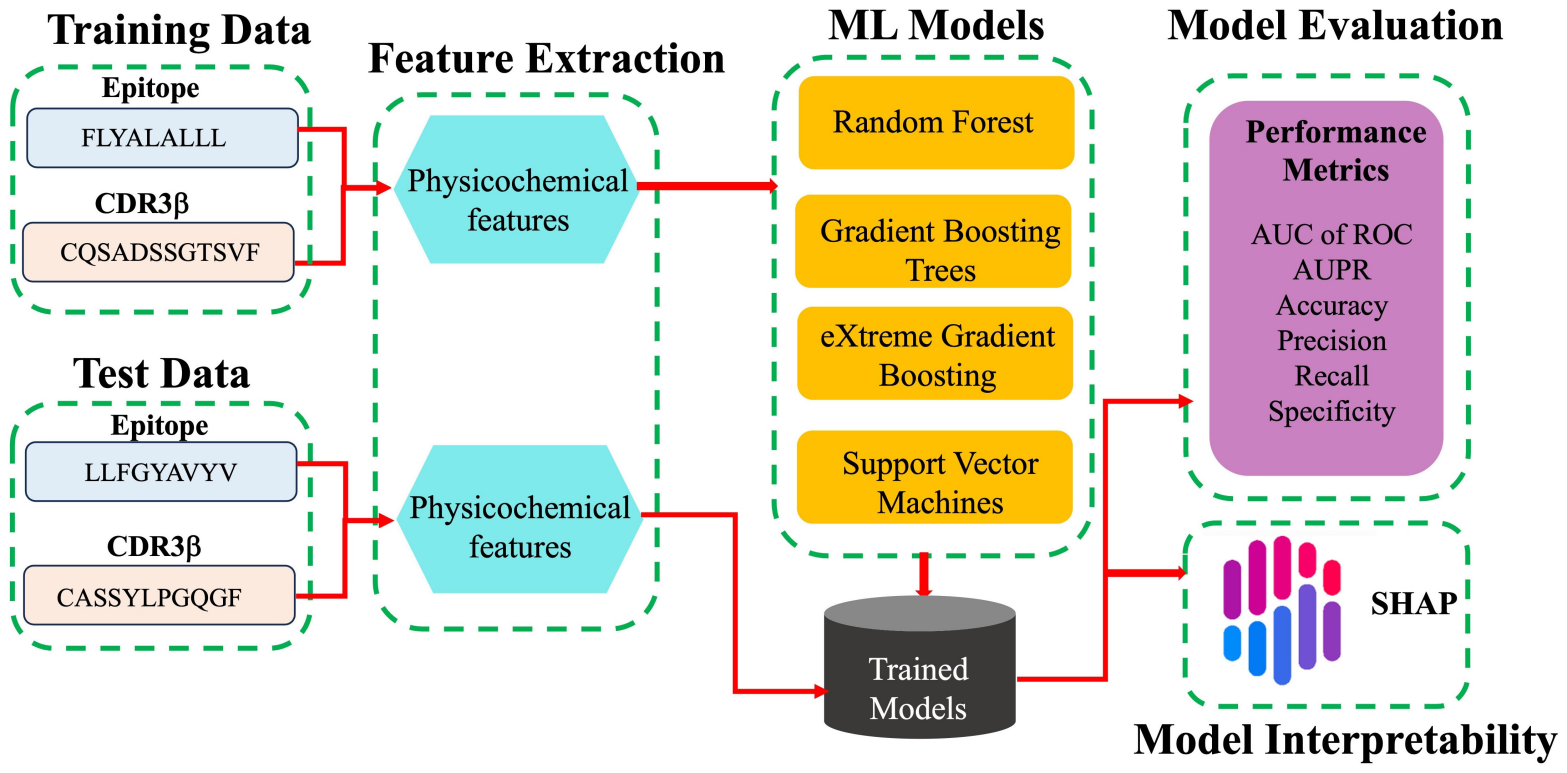
Schematic work flow of ML modeling. For each pair of TCR CDR3β and epitope sequences in the training data, labelled as binding and non-binding, physicochemical features are calculated and provided as input features for the different ML models tested. The trained models predict whether or not given CDR3β and epitope sequences of the test data bind. Models are evaluated based on a variety of performance metrics and are interpreted using SHAP analysis.

The modeling workflow is shown as a schematic in **Figure 1**. The binary classification is the prediction of binding or non-binding. The performance of the models was evaluated in terms of the AUC of ROC, the accuracy, the precision (the ratio of true binders to the total number predicted to be binders, also called positive predictive value), the recall (also called the sensitivity or true positive rate), the specificity (true negative rate), and the F1 score. These terms are defined for convenience in the SI. The AUC of ROC, which is most commonly reported, illustrates the trade-off between the recall (true positive rate) and the specificity (true negative rate), quantifying the ability of the model to distinguish between the two classes. A classifier with perfect performance yields an AUC of ROC of 1.0, while a random classifier would yield an AUC of 0.5 or less.

**Figure 2:**
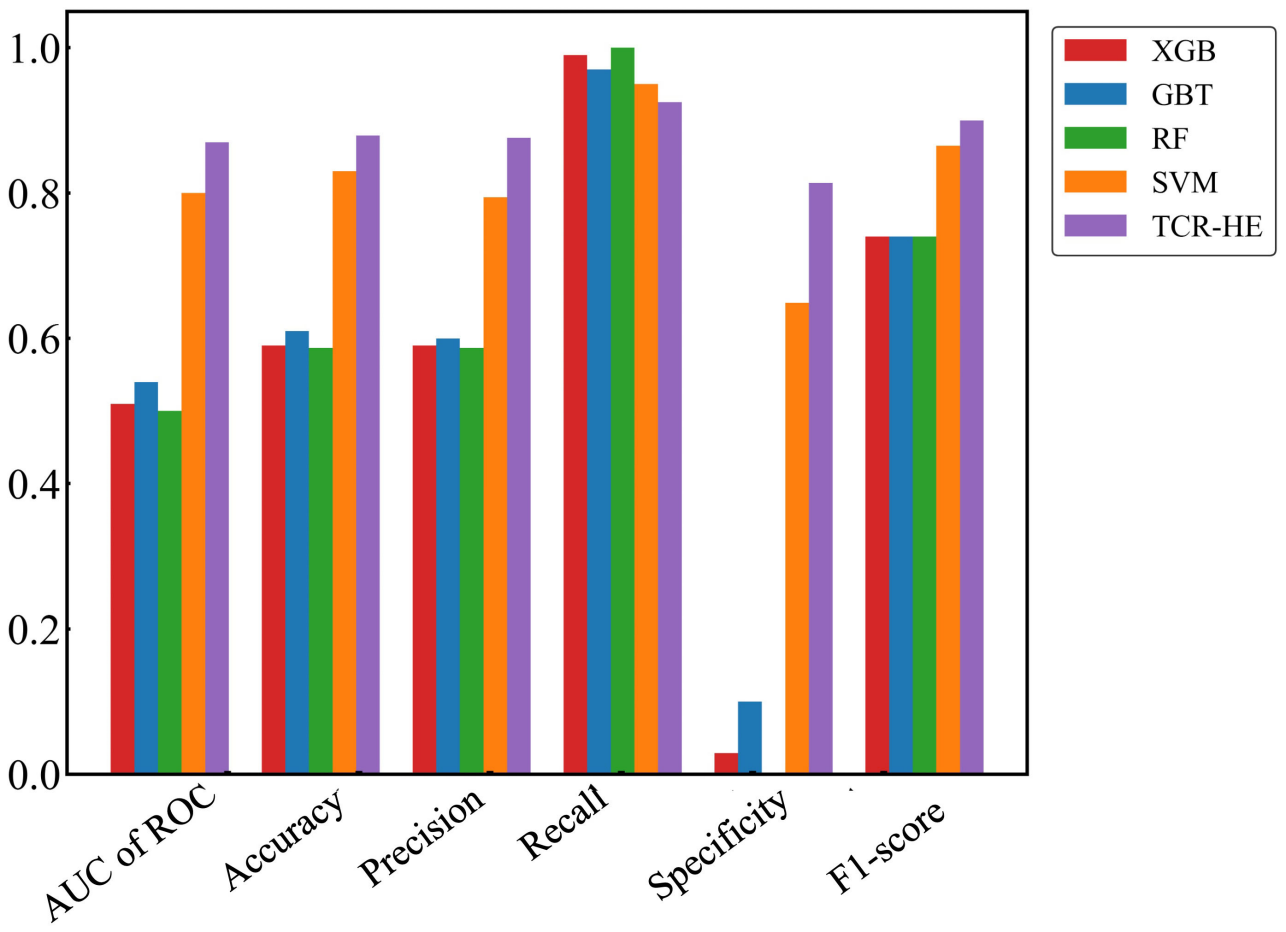
Performance metrics for epitope hard split test set of ML models. Random Forest (RF), Gradient Boosting trees (GBT), eXtreme Gradient Boosting (XGB), Support Vector Machines (SVM) and SVM with uncorrelated features (TCR-HE).

We first present ML performance results on epitope hard split data, i.e., in which all epitopes in the test set are unseen. The performance metrics on the test set for RF, GBT, XGB and SVM are presented in **Table 2** and **Figure 2**. Taking all the metrics into consideration, it is evident that the ensemble models RF, GBT and XGB do not perform particularly well, especially for the AUC of ROC and specificity. Only in terms of recall and the F1-score do these models perform somewhat decently; however, it should be noted that the F1-score, a composite metric that is a function of both precision and recall, is elevated here only by the recall, with the precision being relatively poor. In contrast, for the model trained with SVM, the performance is much better across the board, with the AUC of ROC, accuracy, and precision—all poor in the ensemble tree models—improved dramatically. The AUC of ROC is 0.80, an improvement on previously reported efforts.

**Table 2:** Comparison of ML models for epitope hard split test set.

Model	AUC of ROC	TP	TN	FP	FN	Accuracy	Precision	Recall	Specificity	F1-score
XGB	0.51	31859	654	21817	104	0.597	0.593	0.996	0.029	0.744
GBT	0.54	31248	2329	20142	715	0.617	0.608	0.977	0.104	0.749
RF	0.5	31963	0	22471	0	0.587	0.587	1.00	0.0	0.739
SVM	0.80	30403	14593	7878	1560	0.826	0.794	0.951	0.649	0.865
TCR-HE	0.87	29567	18297	4174	2396	0.879	0.876	0.925	0.814	0.9

TP, number of true positives; TN, number of true negatives; FP, number of false positives; FN, number of false negatives. TCR-HE has correlations removed.

In an attempt to further improve the SVM performance, we examined correlations between pairs of features. We found that by removing one of any pair that had a correlation > 0.8 (we selected the feature to be removed from the correlated pair at random), the model’s performance improved, leading to a model (named “TCR-HE”; “HE”=“hard epitope” split) with an AUC of ROC of 0.87 as well as improving most other statistical metrics. This model has very high sensitivity, i.e., a large fraction of the true positives is predicted positive. The specificity (true negative rate) is also high (this metric has improved to great extent upon consideration of uncorrelated features). To further test the robustness of the model’s performance, we conducted three other epitope hard splits while also stress testing by introducing imbalanced numbers of negative and positive data points. The resulting performance metrics are detailed in the supporting information (**Supplementary Table 2**). The AUC for these scenarios ranged between 0.72 and 0.853, maintaining high, if not quite as high, performance.

Next, we trained the model that uses SVM and the set of uncorrelated features with a hard split of the TCR CDR3βs (a model referred to as TCR-Hβ). The results on the independent test set, provided in **Table 3**, show that again the model performs very well, with an AUC of ROC of 0.92 with other metrics also being high. Again, to test dataset robustness we conducted three additional hard splits for TCR CDR3β and also three for the strict split (**Supplementary Table 2**). The performance metrics consistently demonstrated an AUC of ROC of 0.92 for the different TCR hard splits. This model appears to be particularly stable to dataset variation, which may arise from there being ~150k unique TCR sequences in the dataset as compared to ~800 unique epitope sequences.

**Table 3:** Performance metrics of TCR-H trained and tested on TCR hard split (TCR-Hβ), strict split (TCR-HβE) and random split (TCR-RS).

Model	AUC of ROC	TP	TN	FP	FN	Accuracy	Precision	Recall	Specificity	F1-score
TCR-Hβ	0.92	18760	28780	766	2994	0.93	0.96	0.86	0.97	0.91
TCR-HβE	0.89	29570	19143	3328	2393	0.89	0.898	0.92	0.85	0.91
TCR-RS	0.92	18382	28771	691	3045	0.93	0.96	0.86	0.98	0.91

These results represent the ML trained on the uncorrelated features.

**Figure 3:**
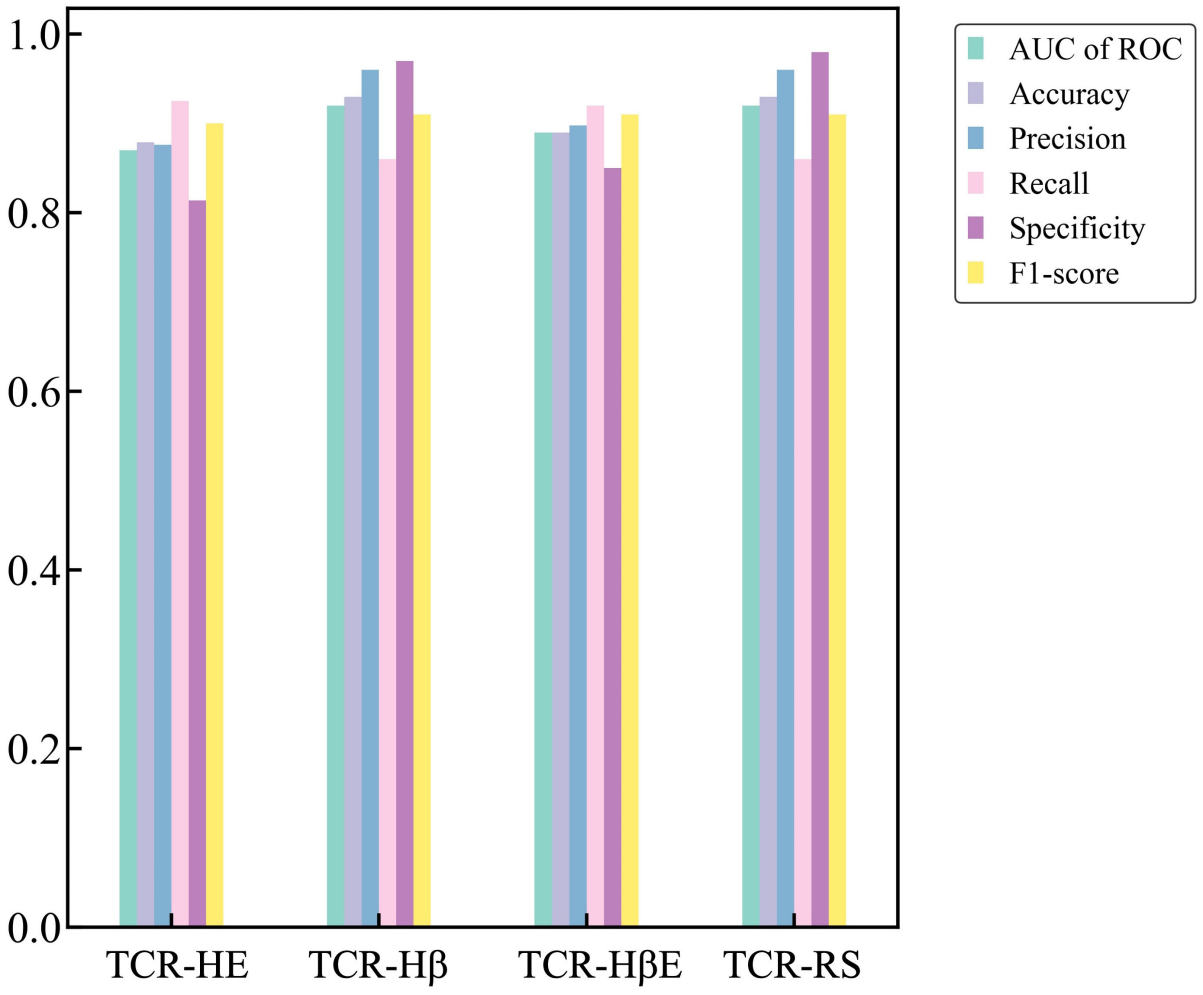
Performance metrics on the independent test sets for the TCR-HE (epitope hard split), TCR-Hβ (TCR hard split), TCR-HβE (strict split) and TCR-RS (random split).

Finally, we report metrics on the test set that perhaps most closely represents a typical situation that will be found in general applications – the strict split, TCR-HβE. This was found to also be very successful, with an AUC of ROC of 0.89 and the other metrics again also high (**Table 3**). The strict splits with imbalanced data yielded AUC of ROC values ranging between 0.71 and 0.83 (**Supplementary Table 2**), again with excellent performance. **Figure 3** shows all the performance metrics on the independent test sets of epitope hard split, TCR hard split, strict split and random split. All the performance metrics are observed to be equal to, or greater than, 0.8.

For completeness, and to compare against what has been typically performed in previous predictive model development studies, we also performed a benchmark of TCR-H using the commonly employed random split (TCR-RS). We recall that in a random split the entire dataset is split randomly into training and test sets and that, because the dataset contains instances where a TCR or epitope is represented multiple times, a random split of the data can lead to the same epitope or TCR being in both the training and test sets. For TCR-RS, the AUC of ROC of TCR-H model is 0.92 (**Table 3**) which is comparable to previously published methods. Additionally, three other TCR-RS random splits employed also showed an AUC of ROC of 0.92, again indicating model robustness.

### Comparison with previous models

**Figure 4:**
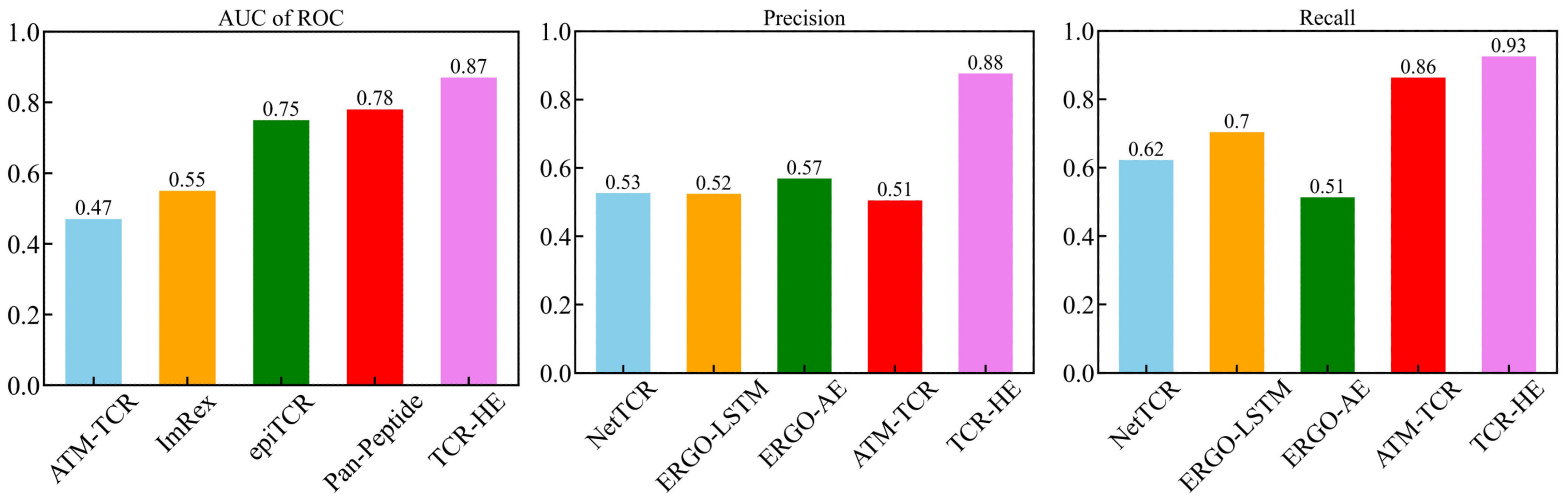
AUC of ROC, Precision and Recall of TCR-HE compared with that of previously reported epitope hard split models (21, 23, 25, 36).

The field of computational epitope-TCR binding prediction is very active and rapidly evolving. However, it is incumbent on us to compare the performance of TCR-H with models hitherto reported in the literature (23, 25, 26, 36, 37, 64). Metrics other than the AUC of ROC are important in potential applications of these methodologies but are not always calculated. However, data exist allowing us to compare the performance of TCR-HE with some reported data for epitope hard-split AUC of ROC, precision, and recall. **Figure 4** shows metrics for various methods taken from (23) all of which were evaluated on the same independent epitope hard split data set, together with the present TCR-HE results. TCR-HE exhibits the highest values in all three metrics. Some methods, including Pan-Peptide and epiTCR, have, in some epitope hard-split tests, shown significant improvement over random, with AUC of ROC scores of ~0.75. Notwithstanding, TCR-HE performed significantly better. Furthermore, the pitfalls of negative data bias in the TCR epitope specificity challenge have recently been emphasized, with, for example, the performance of Pan-peptide reduced to random (AUC of ROC = 0.49) depending on the method of choosing the negative data points (31).

Published data on TCR hard splitting and strict splitting are rarer. However, Cai et al. (2022) (23) reported TCR hard split metrics for four models (NetTCR, ERGO-LSTM, ERGO-AE and ATM-TCR), with the AUC of ROC varying between 0.72 and 0.77, recall between 0.71 and 0.77 and precision between 0.63 and 0.70, again lower than the values obtained with TCR-Hβ (**Table 2**) whereas TITAN (30) achieved an AUC of ROC of 0.87 using a semi-frozen pretrained model with augmentation. A strict split employed by TITAN achieved an AUC of ROC of 0.62.

### Explainable AI: SHAP analysis for model interpretation

**Figure 5:**
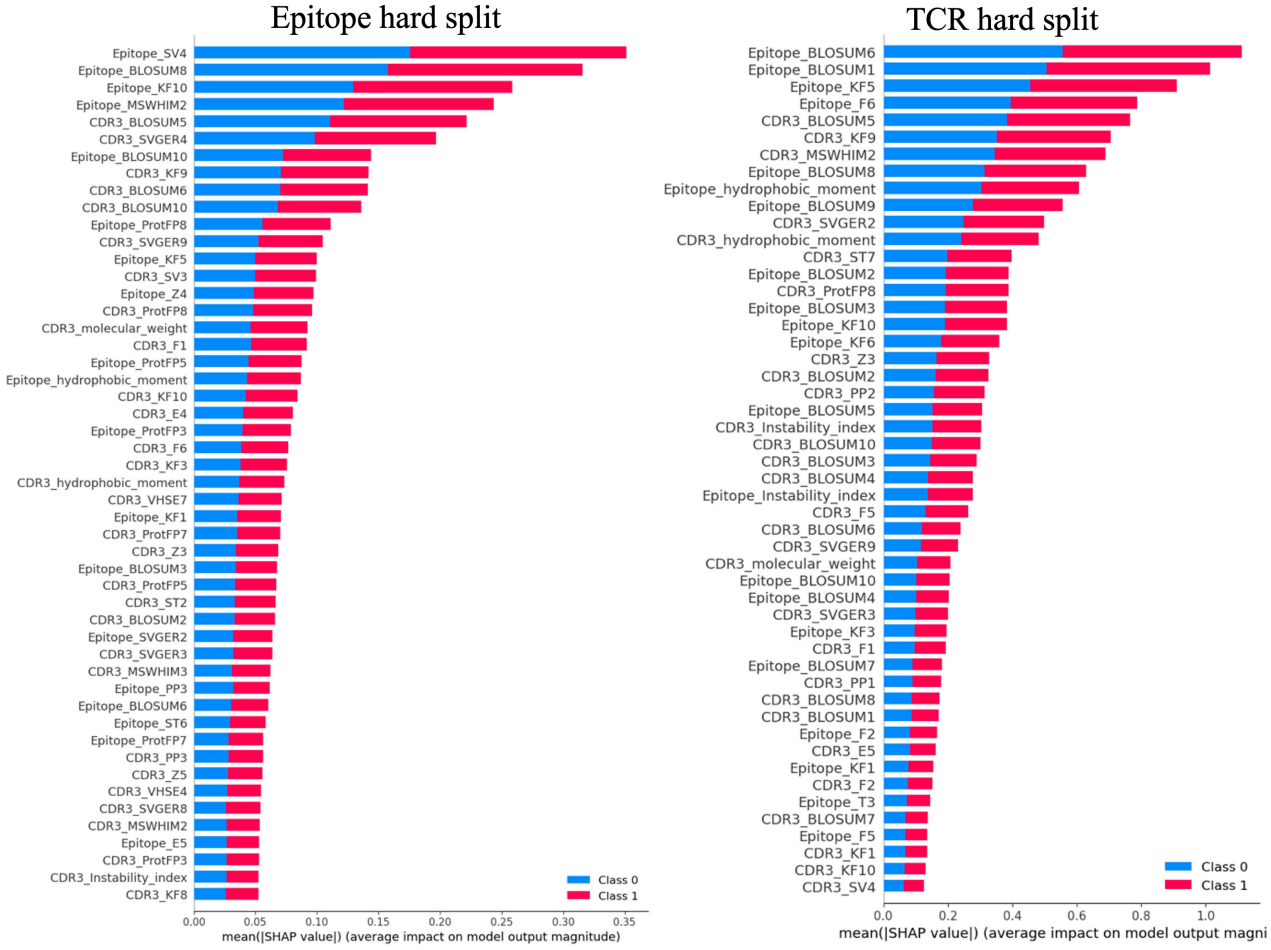
Representative summary plots of SHAP for Epitope split and TCR split showing top 50 features contributing to the model predictions. The length of the horizontal bar corresponding to each significant feature represents the magnitude of its SHAP value, while the color indicates the direction of its impact. Red bars denote higher predicted probabilities for the positive class, whereas blue bars represent the negative class. Longer bars against a feature indicate a greater impact on the model’s prediction.

**Figure 5** depicts representative summary plots of the SHAP analysis for the epitope and TCR hard splits, revealing the importance of the features. The figure displays the top 50 features. Interestingly, for all models with different hard splits, a similar list of features contributing most to the model predictions was found. These include hydrophobic moments, molecular weights, instability index, BLOSUM indices, Kidera factors, SVGER, FASGAI vectors, MSWHIM scales, ProtFP descriptors, and the Z-scales of both epitope and CDR3β sequences. This finding was observed to be consistent across different training and test splits performed in the present study. The significance of BLOSUM indices is consistent with the relative success of previously reported models based solely on these substitution matrices (21, 23, 25, 64). However, the improved results in the present model underscore the usefulness of considering additional physico-chemical features as well as composite features to enhance model performance. Specifically, for both epitopes and CDR3β sequences Kidera factors KF3, KF5, KF7, KF9, and KF10, corresponding to preferences for structures that are extended, double-bend or flat, the pK-C, and the surrounding hydrophobicity, respectively, are found to be important. Also FASGAI vectors F1, F2, F4, F5, and F6, corresponding to the hydrophobicity, alpha and turn propensities, compositional characteristics (F4), local flexibility (F5), and electronic properties (F6) of the sequences, and MSWHIM scales differentiating Asp/Glu and Arg/Lys are also found to be significant.

The CDR3β molecular weight, identified as an important feature, suggests that the length of the CDR3β sequence may play a role in epitope recognition and binding, aligning with previous observations indicating length complementarity between CDR3β and epitope sequences (1). The feature corresponding to hydrophobicity, playing a crucial role in binding prediction, supports previous studies suggesting that enriched hydrophobicity of epitopes aids T cell receptors in discriminating immunogenic epitopes and self-peptides (65). Furthermore, features corresponding to extended structure preference, local flexibility, and electronic properties contributing to binding prediction are consistent with both structural and dynamical properties being important for binding.

## Discussion

Building on our previous work on 3D structural and physicochemical approaches to studies of MHC:epitope:TCR interactions (9, 11), we present here a set of ML models capable of predicting TCR and epitope binding for cases in which epitopes and/or TCR sequences are not included in data used to train the model. The approach is even found to work well for ‘strict split’ cases in which neither the epitopes nor the TCR sequences in the test set are in the training set. Our approach to this was stepwise, in which the performance of TCR-H was first tested on hard splits of epitopes (TCR-HE) and TCRs (TCR-Hβ). Both of these produced excellent metric results that were robust to training/testing dataset variation, with the TCR-Hβ being particularly impressive. The strict split TCR-HβE achieved an AUC of ROC of 0.89, with other metrics such as precision and recall also excellent.

AI/ML methods can be subject to biases in the data sets used for training and testing. We therefore paid special attention here to the quality of the datasets used. In particular, we only considered experimentally validated data, as opposed to using negative data that is randomly generated. Notwithstanding, the testing of a given model on different specific epitope hard split data sets can lead to significantly different AUC values. For example, **Supplementary Figure 1** shows significant dataset dependency of previously reported models on epitope hard split AUC of ROC data. Here, we examined the performance of the TCR-H class of models upon dataset variation. We find good performance even when the test data are burdened with significant positive and negative data imbalance. Notwithstanding, although the dataset used has >200,000 entries, as for most models the approach taken here will need continual further testing and refining. In particular, the addition of experimental data expanding the number of unique epitopes would be expected to further improve model performance. Furthermore, as stated in (64) there is room for improvement in the accuracy of the negative experimental data generated. When considering the unseen elements within the test datasets, it is possible that some epitopes/TCRs are unseen yet still share some sequence similarity – this might lead to enhanced performance metrics, in contrast to cases where epitope/TCR sequences are entirely dissimilar and unseen in the test dataset. Regardless of the epitopes or TCRs sequences with or without sequence similarity, our results showcase that the performance is much higher than that of previous studies.

The excellent performance of the TCR-H models suggests that supervised SVM, when applied to a feature set consisting of physicochemical features derived from whole sequences of TCR CDR3βs and epitopes, may be able to capture complex feature dependencies and interdependencies underlying accurate binding classification. It is interesting that ensemble tree-based methods did not perform as well as SVM, suggesting that to achieve accurate binding prediction, a complex function of the full set of features is advantageous, as opposed to an ensemble of simple, nonparametric “functions”, as represented by the simple individual trees. This was also evident from our analysis of two different dimension reduction techniques in the feature space, namely, PCA (Principal Component Analysis) and t-SNE (t-distributed Stochastic Neighbor Embedding). The datapoints were projected onto the plane formed by the two major reduced dimensions, and the datapoints were labeled according to their class. Both these dimension reduction techniques indeed showed that there is some overlap between the two classes (shown in the **Supplementary Figure 2**) and may explain why the classes are separable using only a supervised ML algorithm that performs a nonlinear projection of the feature vectors.

Also, the utilization of SHapley Additive exPlanations (SHAP) has provided insight into the significant physico-chemical features of both TCRs and epitopes that are pivotal in determining the binding of TCRs to epitopes. Furthermore, the SHAP analysis reveals the importance of pure physico-chemical features as well as composite features that are themselves functions of these pure features, the latter revealing the fact that there is a complex interdependency among physico-chemical properties that govern binding.

For general applicability, a TCR:epitope recognition model must be able to predict binding for TCRs and epitopes for any given TCR:epitope pair. This should include cases in which neither the TCRs nor the epitopes queried are in the training set nor are close in sequence to members in the training set, i.e., the “strict split” scenario. The success in this endeavor reported here may indicate that algorithms are beginning to learn general principles of TCR:epitope recognition. TCR-H is a significant step in this direction, and towards making the vast universe of potential epitope and TCR sequences amenable to the computational prediction of functional TCR/epitope pairs.

At this point, it is incumbent upon us to recognize certain caveats of our model that will inform future work. First, our model is strictly a binary prediction and therefore cannot quantitatively predict the affinity. Furthermore, TCR-pMHC interactions have affinities in micromolar range (6, 7) and are therefore very weak, rendering development of quantitatively accurate regression-based ML models challenging. Affinity prediction may be useful in the future but would require a dedicated regression-based model trained/tested only on data points for which affinities are reported. In the training data used here affinities were only reported for a very small fraction of the total number of datapoints (572 out of ca. 100,000 datapoints) and then only for formation of the ternary complex. Here we train a classification model to make a bind vs. does not bind prediction of TCR:epitope pairs.

Aside from the limitation listed above, TCR binding is modulated and finely tuned by the microenvironmental context. One such effect is the CD8 cell-surface glycoprotein. Class I interactions can be stabilized by CD8 and taking this into account in future studies may be useful once the appropriate experimental data become available (8). Furthermore, it has been realized that the TCR is much more than a binary receptor, as it must interpret and respond to a range of ligand strengths throughout T-cell development and phenotypic differentiation. The TCR is highly specific (66–70), while remaining versatile, allowing TCR interaction with a range of pMHC, corresponding to virtually any foreign antigen. Moreover, the TCR-pMHC complex is modulated by its microenvironmental context, including soluble factors (e.g., cytokines, growth factors, and nutrients) and sessile molecular cues in the tissue microenvironment, facilitating a range of diverse and specific responses by T-cells (71). In addition to these effects, the affinity of the TCR-pMHC complex is also modulated by external forces by virtue of the fact that the TCR is mechanosensitive, a property arising from the motility of lymphocytes (6, 72–76). The above properties enable T cells to tailor responses to pathogens while avoiding autoimmune disease. Clearly the delineation of such effects is way beyond the abilities of a simple CDR3beta:epitope machine learning model as is presented here. However, there is some suggestion from our results that elements of the fundamental epitope:receptor interaction, aside from the context-specific modulatory effects, are indeed being captured.

## Code availability

TCR-H is available on Github at https://github.com/rajitha-tatikonda/TCR-H.

## Data Availability

The original contributions presented in the study are included in the article/**Supplementary Material**, further inquiries can be directed to the corresponding authors.
