# Supplementary material for: TCR-H: explainable machine learning prediction of T-cell receptor epitope binding on unseen datasets
Source: Front Immunol. 2024 Aug 16;15:1426173. doi: 10.3389/fimmu.2024.1426173 (PMC11361934; doi:10.3389/fimmu.2024.1426173)
Supplement: Supplementary file 1 [file DataSheet1.docx]

**Supporting Information**

**TCR-H:**

**Explainable Machine Learning Prediction of T-cell Receptor Epitope Binding on Unseen Datasets**

**Dataset Details.**

In Table S1 we present the categorized numbers of data in the various datasets used.

Table S1: Categorized dataset numbers. These training/testing data are used to train/test the models whose performance is given in Table 1 and 2 of the Main Text.

Epitope Hard Split:

| Training dataset | | | Test dataset | | |
| --- | --- | --- | --- | --- | --- |
| Positive data | Negative data | Unique peptides | Positive data | Negative data | Unique peptides |
| 75413 | 124598 | 790 | 31963 | 22471 | 65 |

TCR Hard Split:

| Training dataset | | | Test dataset | | |
| --- | --- | --- | --- | --- | --- |
| Positive data | Negative data | Unique CDR3βs | Positive data | Negative data | Unique CDR3βs |
| 85621 | 117523 | 124098 | 21754 | 29546 | 31025 |

Strict Split:

| Training dataset | | | | Test dataset | | | |
| --- | --- | --- | --- | --- | --- | --- | --- |
| Positive data | Negative data | Unique CDR3βs | Unique peptides | Positive data | Negative data | Unique CDR3βs | Unique peptides |
| 65826 | 83697 | 105011 | 739 | 31963 | 22471 | 50112 | 65 |

**Performance Metric Definitions.**

The performance model metrics are defined as follows:

Accuracy (ACC) is the proportion of correctly classified instances:

Accuracy= $\frac{Number of Correct Predictions}{Total Number of Predictions}$

1

Precision (also called Positive Predictive Value) is the proportion of true positives among instances predicted as positive:

Precision= $\frac{True Positives}{True Positives+False Positives}$

​ 2

Recall (also called Sensitivity or True Positive Rate) is the proportion of true positives among actual positive instances:

Recall= $\frac{True Positives}{True Positives+False Negatives}$

3

​

F1 Score is the harmonic mean of precision and recall:

F1 Score=$2\times$ $\frac{Precision \times Recall}{Precision+Recall}$

4

​

Specificity (True Negative Rate) is the proportion of true negatives among actual negative instances:

Specificity=$\frac{True Negatives}{True Negatives + False Positives}$ 5

In Table S2 we present the performance metrics of hard splits, strict splits and random splits obtained when varying the balance of positive and negative data as described in the main text. These ML performance metrics are obtained using the uncorrelated feature set.

Table S2: Performance metrics when varying the balance of positive and negative data.

| **Model** | **AUC of ROC** | **TP** | **TN** | **FP** | **FN** | **Accuracy** | **Precision** | **Recall** | **Specificity** | **F1-score** |
| --- | --- | --- | --- | --- | --- | --- | --- | --- | --- | --- |
| **TCR-HE** | 0.85 | 12487 | 19446 | 3087 | 2300 | 0.855 | 0.801 | 0.844 | 0.863 | 0.82 |
|  | 0.72 | 1361 | 1624 | 1785 | 46 | 0.619 | 0.432 | 0.967 | 0.476 | 0.59 |
|  | 0.79 | 4470 | 164 | 91 | 294 | 0.923 | 0.98 | 0.938 | 0.64 | 0.958 |
|  | | | | | | | | | | |
| **TCR-Hβ** | 0.92 | 18760 | 28780 | 766 | 2994 | 0.926 | 0.96 | 0.862 | 0.974 | 0.908 |
|  | 0.92 | 18362 | 28524 | 742 | 3033 | 0.925 | 0.96 | 0.858 | 0.974 | 0.906 |
|  | 0.92 | 18309 | 28660 | 824 | 3058 | 0.923 | 0.956 | 0.856 | 0.972 | 0.904 |
|  | | | | | | | | | | |
| **TCR-HβE** | 0.83 | 13714 | 16406 | 6127 | 1073 | 0.807 | 0.691 | 0.927 | 0.728 | 0.792 |
|  | 0.71 | 1355 | 1574 | 1835 | 52 | 0.608 | 0.424 | 0.963 | 0.461 | 0.589 |
|  | 0.77 | 4423 | 160 | 95 | 341 | 0.913 | 0.978 | 0.928 | 0.627 | 0.953 |
|  | | | | | | | | | | |
| **TCR-RS** | 0.92 | 18358 | 28827 | 672 | 3032 | 0.93 | 0.96 | 0.86 | 0.98 | 0.91 |
|  | 0.92 | 18327 | 28787 | 698 | 3077 | 0.93 | 0.96 | 0.86 | 0.98 | 0.91 |
|  | 0.92 | 18246 | 28921 | 659 | 3063 | 0.93 | 0.97 | 0.86 | 0.98 | 0.91 |


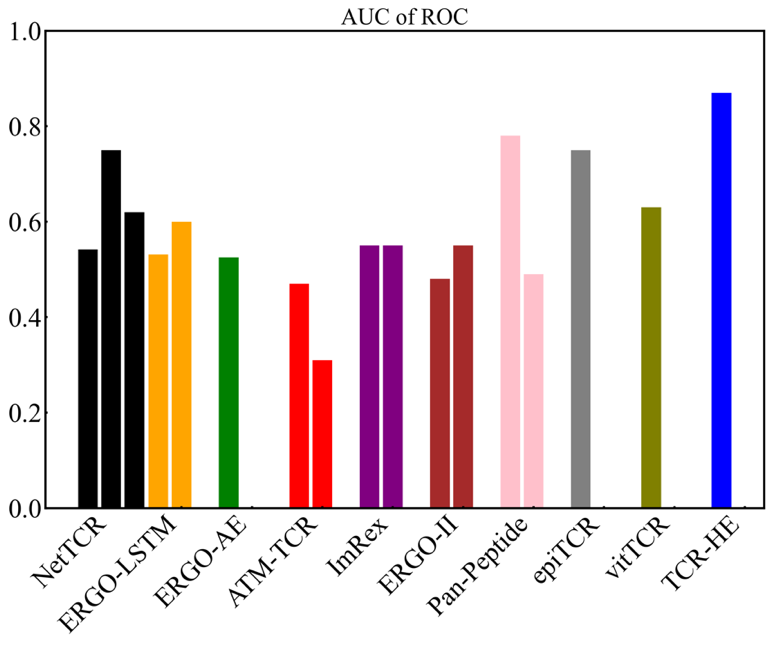


Figure S1. Epitope Hard Split AUC of ROC data with TCR-HE and various previously-reported models. Each bar represents the given model with the same training set but tested on a specific hard split data set. Data taken from (Cai et al., 2022; Gao, Gao, Fan, et al., 2023b; Grazioli et al., 2022; Jiang et al., 2023; Pham et al., 2023)

**
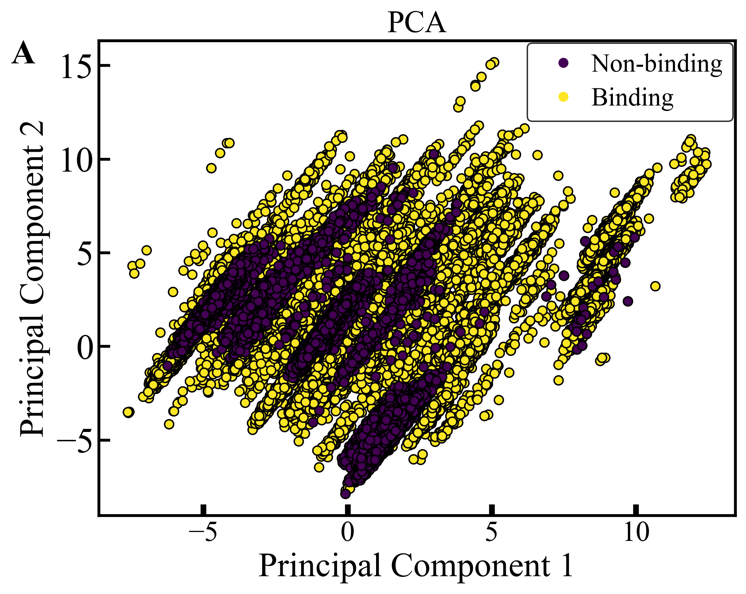

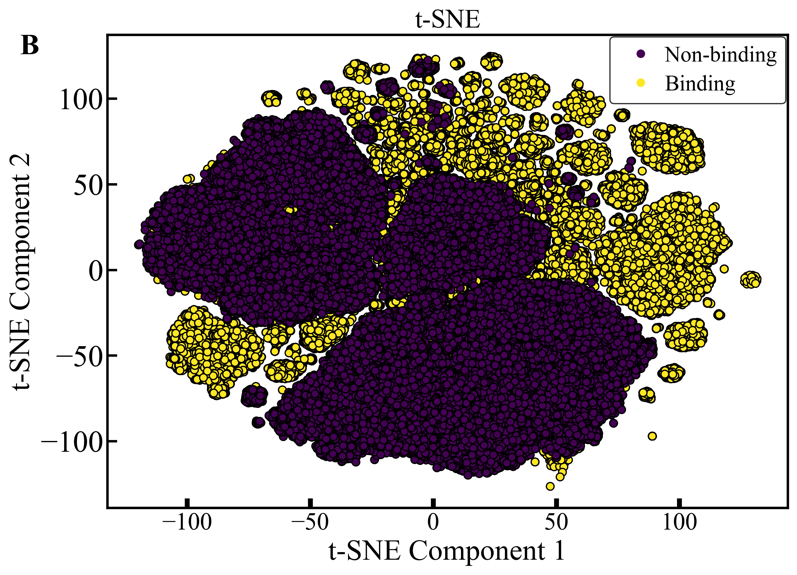
**

Fig S2**.** A) PCA (Principal Component Analysis) and B) t-SNE (t-distributed Stochastic Neighbor Embedding) dimensionality reduction is shown with datapoints projected onto the two major reduced dimensions with classes color coded.
